# Marginal evidence for cosmic acceleration from Type Ia supernovae

**DOI:** 10.1038/srep35596

**Published:** 2016-10-21

**Authors:** J. T. Nielsen, A. Guffanti, S. Sarkar

**Affiliations:** 1 Niels Bohr Institute, University of Copenhagen, Blegdamsvej 17, Copenhagen 2100, Denmark; 2 Dipartimento di Fisica, Università degli Studi di Torino, via P. Giuria 1, I-10125 Torino, Italy; 3 Rudolf Peierls Centre for Theoretical Physics, University of Oxford, 1 Keble Road, Oxford OX1 3NP, UK

## Abstract

The ‘standard’ model of cosmology is founded on the basis that the expansion rate of the universe is accelerating at present — as was inferred originally from the Hubble diagram of Type Ia supernovae. There exists now a much bigger database of supernovae so we can perform rigorous statistical tests to check whether these ‘standardisable candles’ indeed indicate cosmic acceleration. Taking account of the empirical procedure by which corrections are made to their absolute magnitudes to allow for the varying shape of the light curve and extinction by dust, we find, rather surprisingly, that the data are still quite consistent with a constant rate of expansion.

In the late 1990’s, studies of Type Ia supernovae (SN Ia) showed that the expansion rate of the universe appears to be accelerating as if dominated by a cosmological constant^1^,^2^,^3^. Since then supernova cosmology has developed rapidly as an important probe of ‘dark energy’. Empirical corrections are made to reduce the scatter in the observed magnitudes by exploiting the observed (anti) correlation between the peak luminosity and the light curve width and the colour^4^,^5^. Other such correlations have since been found e.g. with the host galaxy mass^6^ and metallicity^7^. Cosmological parameters are then fitted, along with the parameters determining the light curves, by simple *χ*^2^ minimisation^1^,^8^,^9^,^10^,^11^. This method has a number of pitfalls as has been emphasised earlier^12^,^13^.

With ever increasing precision and size of SN Ia datasets, it is important to also improve the statistical analysis of the data. To accomodate model comparison, previous work^14^,^15^,^16^ has introduced likelihood maximisation. In this work we present an improved maximum likelihood analysis, finding rather different results.

## Supernova Cosmology

There are several approaches to making SN Ia ‘standardiseable candles’. The different philosophies lead to mildly different results but the overall picture seems consistent^17^. In this paper we adopt the widely used approach of ‘Spectral Adaptive Lightcurve Template 2′ (SALT2)^18^,^19^ wherein the SN Ia are standardised by fitting their light curve to an empirical template, and the parameters of this fit are used in the cosmological analysis. (A more comprehensive statistical model of light curves spanning optical through near-infrared data has subsequently been constructed in a hierarchical Bayesian framework^20^). Every SN Ia is assigned three parameters, one being 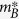, the apparent magnitude at maximum (in the rest frame ‘*B*-band’), while the other two describe the light curve shape and colour corrections: *x*_1_ and *c*. The distance modulus is then taken to be:


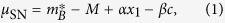


where *M* is the absolute magnitude, and *α* and *β* are assumed to be constants for *all* SN Ia. These global constants are fitted along with the cosmological parameters. The physical mechanism(s) which give rise to the correlations that underlie these corrections remain uncertain^21^,^22^. The SN Ia distance modulus is then compared to the expectation in the standard ΛCDM cosmological model:


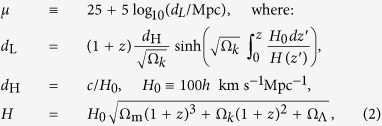


where *d*_L_, *d*_H_, *H* are the luminosity distance, Hubble distance and Hubble parameter respectively, and Ω_m_, Ω_Λ_, Ω_*k*_ are the matter, cosmological constant and curvature density in units of the critical density^3^. There is a degeneracy between *H*_0_ and *M*_0_ so we fix the value of the Hubble parameter today to *h* = 0.7 which is consistent with independent measurements.

## Maximum Likelihood Estimators

To find the maximum likelihood estimator (MLE) from the data, we must define the appropriate likelihood:


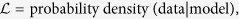


i.e. we have to first specify our model of the data. For a given SN Ia, the true data 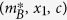 are drawn from some global distribution. These values are contaminated by various sources of noise, yielding the observed values 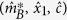. Assuming the SALT2 model is correct, only the true values obey equation (1). However when the experimental uncertainty is of the same order as the intrinsic variance as in the present case, the observed value is *not* a good estimate of the true value. Parameterising the cosmological model by *θ*, the likelihood function can be written as^13^:


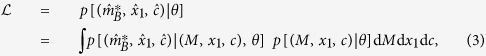


which shows explicitly where the experimental uncertainties enter (first factor) and where the variances of the intrinsic distributions enter (second factor).

**Figure 1:**
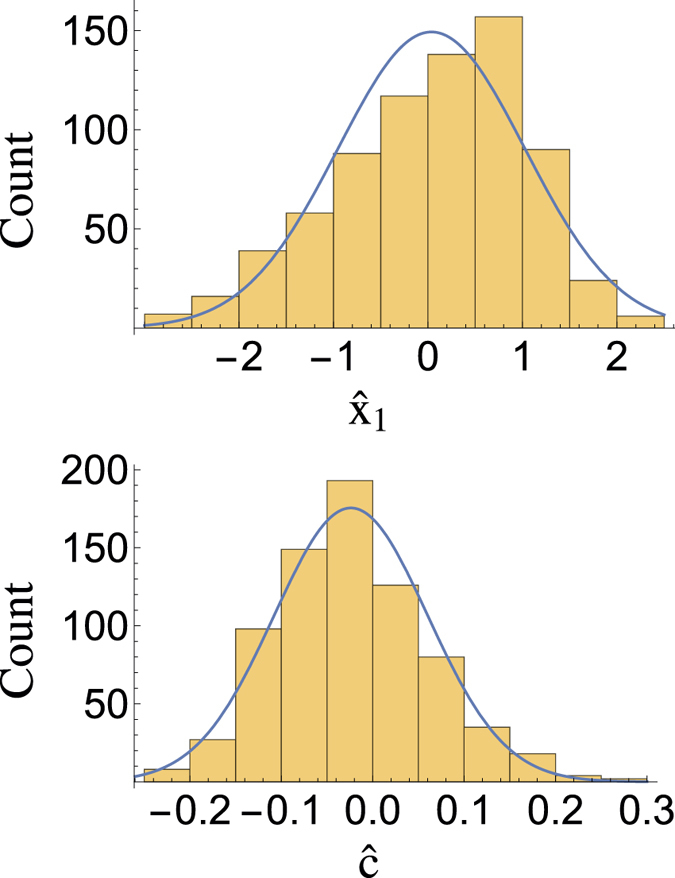
Distribution of the SALT2 stretch and colour correction parameters for the JLA sample^11^ of SN Ia, with our gaussian models superimposed.

Having a theoretically well-motivated distribution for the light curve parameters would be helpful, however this is not available. For simplicity we adopt global, independent gaussian distributions for all parameters, *M*, *x*_1_ and *c* (see Fig. 1), i.e. model their probability density as:


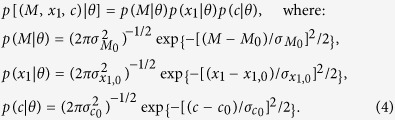


All 6 free parameters 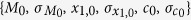 are fitted along with the cosmological parameters and we include them in *θ*. Introducing the vectors *Y* = {*M*_1_, *x*_11_, *c*_1_, … *M*_*N*_, *x*_1*N*_, *c*_*N*_}, the zero-points *Y*_0_, and the matrix 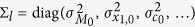, the probability density of the true parameters writes:


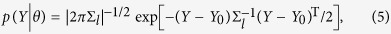


where |…| denotes the determinant of a matrix. What remains is to specify the model of uncertainties on the data. Introducing another set of vectors 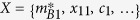, the observed 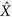, and the estimated experimental covariance matrix Σ_d_ (including both statistical and systematic errors), the probability density of the data given some set of true parameters is:


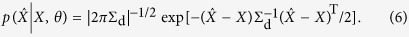


To combine the exponentials we introduce the vector 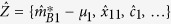 and the block diagonal matrix


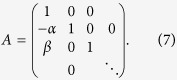


With these, we have 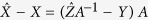 and so 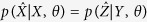. The likelihood is then


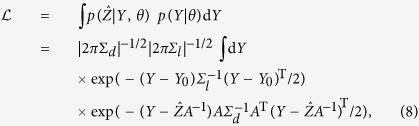


which can be integrated analytically to obtain:


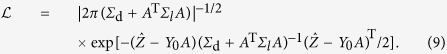


This is the likelihood (equation (3)) for the simple model of equation (4), and the quantity which we maximise in order to derive confidence limits. The 10 parameters we fit are 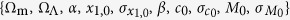. We stress that it is necessary to consider all of these together and Ω_m_ and Ω_Λ_ have no special status in this regard. The advantage of our method is that we get a goodness-of-fit statistic in the likelihood which can be used to compare models or judge whether a particular model is a good fit. Note that the model is not just the cosmology, but includes modelling the distributions of *x*_1_ and *c*.

With this MLE, we can construct a confidence region in the 10-dimensional parameter space by defining its boundary as one of constant 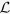. So long as we do not cross a boundary in parameter space, this volume will asymptotically have the coverage probability


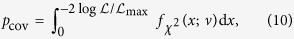


where 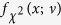 is the pdf of a chi-squared random variable with *ν* degrees of freedom, and 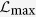 is the maximum likelihood.

To eliminate the so-called ‘nuisance parameters’, we set similar bounds on the profile likelihood. Writing the interesting parameters as *θ* and nuisance parameters as *ϕ*, the profile likelihood is defined as


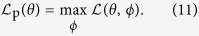


We substitute 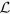 by 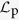 in equation (10) in order to construct confidence regions in this lower dimensional space; *ν* is now the dimension of the remaining parameter space. Looking at the Ω_m_ − Ω_Λ_ plane, we have for 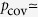  {0.68 (“1*σ*”), 0.95 (“2*σ*”), 0.997 (“3*σ*”)}, the values 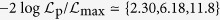 respectively.

### Comparison to other methods

It is illuminating to relate our work to previously used methods in SN Ia analyses. One method^14^ maximises a likelihood, which is written in the case of uncorrelated magnitudes as


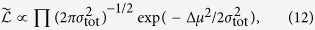


so it integrates over *μ*_SN_ to unity and can be used for model comparison. From Equation (3) we see that this corresponds to assuming *flat* distributions for *x*_1_ and *c*. However the actual distributions of 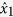 and 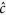 are close to gaussian, as seen in Fig. 1. Moreover although this likelihood apparently integrates to unity, it accounts for only the 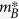 data. Integration over the *x*_1_, *c* data demands compact support for the flat distributions so the normalisation of the likelihood becomes arbitrary, making model comparison tricky.

More commonly used^1^,^8^ is the ‘constrained *χ*^2^’


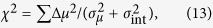


but this cannot be used to compare models, since it is *tuned* to be 1 per degree of freedom for the ΛCDM model by adjusting an arbitrary error *σ*_int_ added to each data point. This has been criticised^12^,^13^, nevertheless the method continues to be widely used and the results presented without emphasising that it is intended only for parameter estimation for the *assumed* (ΛCDM) model, rather than determining if this is indeed the best model.

## Analysis of JLA catalogue

**Figure 2:**
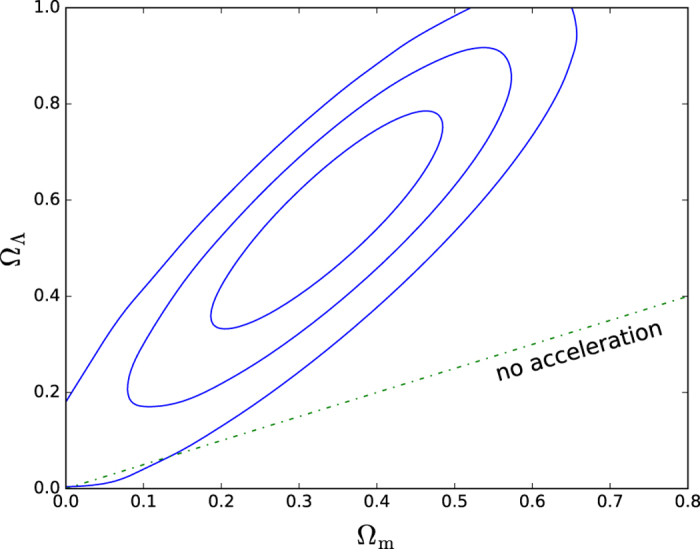
Contour plot of the profile likelihood in the Ω_m_ − Ω_Λ_ plane. We show 1, 2 and 3*σ* contours, regarding all other parameters as nuisance parameters.

**Figure 3:**
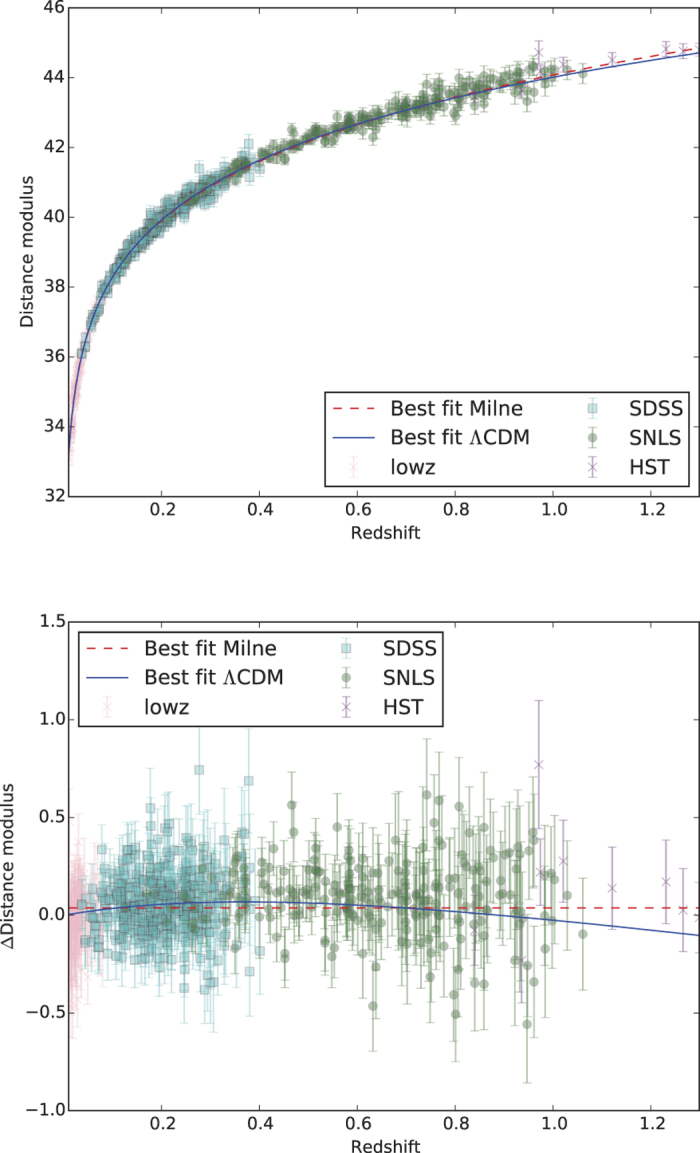
Comparison of the measured distance modulus with its expected value for the best fit accelerating universe (ΛCDM) and a universe expanding at constant velocity (Milne). The error bars include both experimental uncertainties and intrinsic dispersion. The bottom panel shows the residuals relative to the Milne model.

We focus on the Joint Lightcurve Analysis (JLA) catalogue^11^. (All data used are available on http://supernovae.in2p3.fr/sdss_snls_jla/ReadMe.html — we use the covmat_v6.) As shown already in Fig. 1, the distributions of the light curve fit parameters 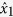 and 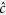 are well modelled as gaussians. Maximisation of the likelihood under specific constraints is summarised in Table 1 and the profile likelihood contours in the Ω_m_ − Ω_Λ_ plane are shown in Fig. 2. In Fig. 3 we compare the measured distance modulus, 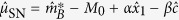 with its expected value in two models: ‘ΛCDM’ is the best fit (Table 1) accelerating universe, while ‘Milne’ is an universe expanding with constant velocity. The error bars are the square root of the diagonal elements of Σ_*l*_ + *A*^T−1^Σ_d_*A*^−1^ so include both experimental uncertainties and intrinsic dispersion. We show also the residuals with respect to the Milne model (which has been raised to take into account the change in *M*_0_).

**Figure 4:**
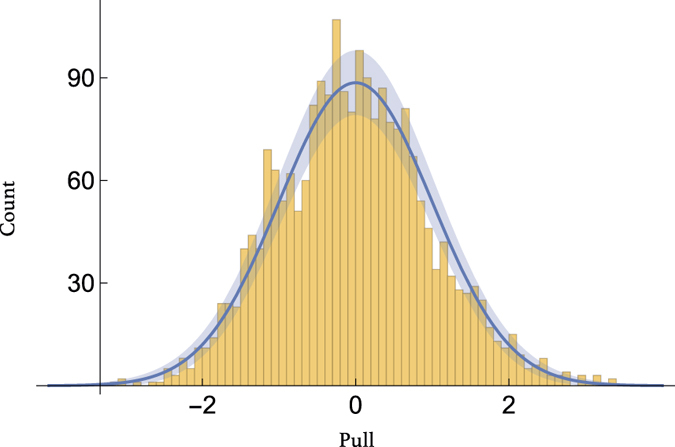
Distribution of pulls (14) for the best-fit model compared to a normal distribution.

To assess how well our Gaussian model for the latent variables describes the data, we show the ‘pull’ distribution in Fig. 4. These are defined as the normalised, decorrelated residuals of the data,


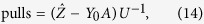


where *U* is the upper triangular Cholesky factor of the covariance matrix Σ_d_ + *A*^T^Σ_*l*_*A*. Performing a K-S test, comparing the pull distribution to a unit variance gaussian gives a p-value of 0.1389.

**Figure 5:**
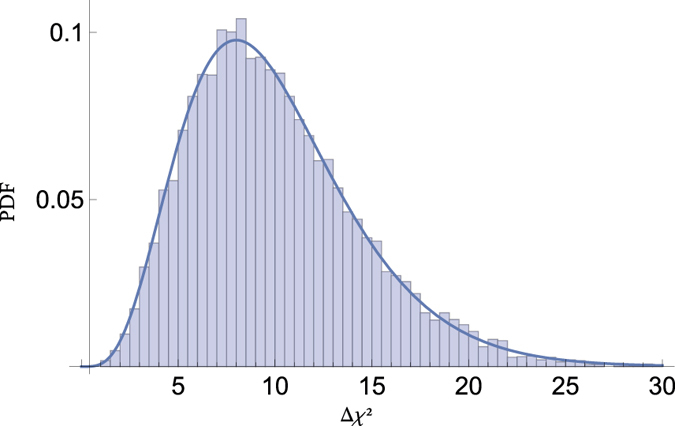
The distribution of the likelihood ratio from Monte Carlo, with a *χ*^2^ distribution with 10 d.o.f. superimposed.

To check the validity of our method and approximations, we do a Monte Carlo simulation of experimental outcomes from a model with parameters matching our best fit (see Table 1). Figure 5 shows the distribution of 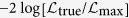, which is just as is expected.

## Discussion

That the SN Ia Hubble diagram appears consistent with an uniform rate of expansion has been noted earlier^16^,^23^,^24^,^25^. We have confirmed this by a statistically principled analysis, using the JLA catalogue of 740 SN Ia processed by the SALT2 method. We find marginal (i.e. 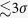) evidence for the widely accepted claim that the expansion of the universe is presently accelerating^3^.

The Bayesian equivalent of this method (a “Bayesian Hierarchical Model”) has been presented elsewhere^13^ and has recently been applied to the same dataset, finding results consistent with ours^26^. We note that a Bayesian consistency test^27^ has been applied (albeit using the flawed ‘likelihood’ (equation 12) and ‘constrained *χ*^2^’ (equation 13) methods) to determine the consistency between the SN Ia data sets acquired with different telescopes^28^. These authors do find inconsistencies in the UNION2 catalogue but none in JLA. This test had been applied earlier to the UNION2.1 compilation finding no contamination, but those authors^29^ *fixed* the light curve fit ‘nuisance’ parameters, so their result is inconclusive. Including a ‘mass step’ correction for the host galaxies of SN Ia^11^ has little effect.

While our gaussian model (4) is not perfect, it appears to be an adequate first step towards understanding SN Ia standardisation. One might be concerned that various selection effects (e.g. Malmquist bias) affect the data. Such effects may not be amenable to our approximate method and are better addressed in a Bayesian approach^26^. We are concerned here solely with performing the analysis in a statistically sound manner to highlight the different conclusion from previous analyses^11^ of the *same* data.

Whether the expansion rate is accelerating or not is a *kinematic* test and it is only for ease of comparison with previous results that we have chosen to show the impact of doing the correct statistical analysis in the ΛCDM framework. In particular the ‘Milne model’ refers here to an equation of state *p* = −*ρ*/3 and should *not* be taken to mean an empty universe. For example the deceleration due to gravity may be countered by bulk viscosity associated with the formation of structure, resulting in expansion at approximately constant velocity even in an universe containing matter but no dark energy^30^. Such a cosmology is not *prima facie* in conflict with observations of the angular scale of fluctuations in the cosmic microwave background or of baryonic acoustic oscillations, although this does require further investigation. In any case, both of these are geometric rather than dynamical measures and do not provide compelling *direct* evidence for a cosmological constant — rather its value is inferred from the assumed ‘cosmic sum rule’: Ω_Λ_ = 1 − Ω_m_ + Ω_*k*_. This would be altered if e.g. an additional term due to the ‘back reaction’ of inhomogeneities is included in the Friedmann equations^31^.

The CODEX experiment on the European Extremely Large Telescope will aim to measure the ‘redshift drift’ over a 10–15 year period to determine whether the expansion rate is really accelerating^32^.

## Methods: Confidence ellipsoids

The confidence ellipsoid is the collection of points 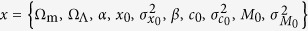, which obey


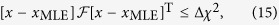


where 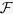 is a symmetric matrix and *x*_MLE_ is the MLE. The enclosed volume is a confidence region with coverage probability corresponding with high precision to the value obtained from Equation (10). The eigenvectors of 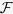 are then the principal axes of the ellipsoid, and the eigenvalues are the inverse squares of the lengths of the principal axes. We approximate this matrix with the sample covariance from the MC of section 3 as 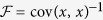.

To make reading the matrix of eigenvectors easier, we round all numbers to 0.1. Thus, we get the following approximate eigenvectors of 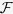, in columns


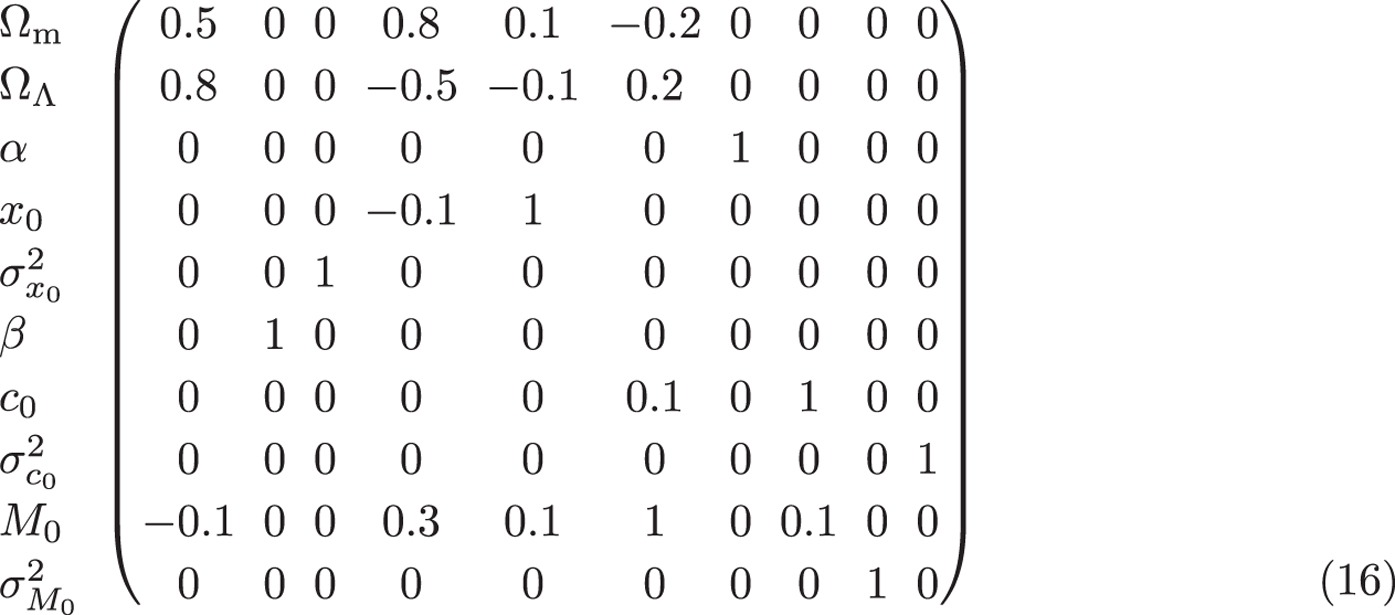


with respective lengths of semi-axes


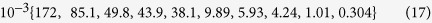


We also list the rounded correlation matrix,


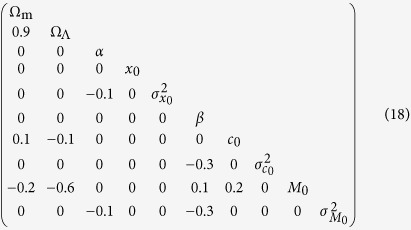


We see that the only pronounced correlations are between Ω_m_, Ω_Λ_ and *M*_0_. This is also apparent from Table 1.

### Code Availability

The code and data used in the analysis are available at: http://dx.doi.org/10.5281/zenodo.34487

## Additional Information

**How to cite this article**: Nielsen, J. T. *et al*. Marginal evidence for cosmic acceleration from Type Ia supernovae. *Sci. Rep.* **6**, 35596; doi: 10.1038/srep35596 (2016).

## Figures and Tables

**Table 1:** Maximum likelihood parameters under specific (boldface) constraints ( 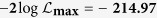 ).

Constraint	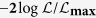	Ω_m_	Ω_Λ_	*α*	*x*_1,0_	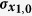	*β*	*c*_0_	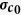	*M*_0_	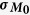
None (best fit)	**0**	0.341	0.569	0.134	0.038	0.932	3.059	−0.016	0.071	−19.052	0.108
Flat geometry	0.147	0.376	**0.624**	0.135	0.039	0.932	3.060	−0.016	0.071	−19.055	0.108
Empty universe	11.9	**0.000**	**0.000**	0.133	0.034	0.932	3.051	−0.015	0.071	−19.014	0.109
Non-accelerating	11.0	0.068	**0.034**	0.132	0.033	0.931	3.045	−0.013	0.071	−19.006	0.109
Matter-less universe	10.4	**0.000**	0.094	0.134	0.036	0.932	3.059	−0.017	0.071	−19.032	0.109
Einstein-deSitter	221.97	**1.000**	**0.000**	0.123	0.014	0.927	3.039	0.009	0.072	−18.839	0.125
